# Fellow-Workers and the Roll of Honour

**Published:** 1914-12-12

**Authors:** 


### ROUND THE WORLD.

[The Editor Invites Early Information and Contributions Marked "Fellow-Workers."]

### Fellow-Workers and the Roll of Honour.

Captain Harry Sherwood Ranken, R.A.M.C., whose bravery in an action at Hautvesne won him the V.C., but also led to his being fatally wounded, was the elder son of a Scottish minister, the Rev. Henry Ranken, of Irvine. He graduated with distinction by taking the M.B., Ch.B. of Glasgow University in 1905, and subsequently was on the resident staff of the Glasgow Western Infirmary and the Brook Fever Hospital. Four years after qualification he decided to enter the Army Medical Department, and succeeded in gaining the first place at the entrance examination, a success which he followed up by winning various important prizes in his probationary course. Captain Ranken did well throughout, and showed himself a keen worker by finding time in the midst of his military duties to take the M.R.C.P. Lond. During the past three or four years he had done important research work in connection with trypanosomiasis, and as a member of the Soudan Government Sleeping Sickness Commission. By his death the R.A.M.C. loses an exceptionally brilliant all-round man, who distinguished himself not only in his professional work, but as a hunter of big game and first-class golfer.

Through the naval action off the coast of Chile and the disaster off Sheerness no fewer than eight naval surgeons lost their lives. Three of these unfortunate officers went down with H.M.S. *Bulwark*—namely, Fleet-Surgeon P. K. Nix, Surgeon W. Miller, and Surgeon R. T. Brotchie.

Mr. Percival Kent Nix, M.B., B.C. Cantab., spent his early student years at Cambridge University and subsequently proceeded to the London Hospital, where he completed his clinical work. Entering the Royal Naval Medical Service the same year, he received promotion in due course and was appointed to the ill-fated *Bulwark* just over two years ago; whilst Mr. William Miller was a Glasgow man who qualified M.B., Ch.B. of his University in 1906 and subsequently held the posts of house-surgeon and house-physician at the Victoria Infirmary, Glasgow.

The medical officers on board H.M.S. *Monmouth* were Staff-Surgeon H. Woods and Surgeon A. J. Tonkinson; whilst the sick berth on H.M.S. *Good Hope* was in charge of Fleet-Surgeon J. J. Walsh, Surgeon F. C. Searle, and Surgeon F. L. J. M. de Verteuil.

Mr. Henry Woods was a Liverpool man and took the M.B., Ch.B., Victoria University, in 1903. Joining the Navy soon after qualification, he became staff-surgeon after eight years' service and had latterly been attached to the Naval Barracks at Devonport. On the outbreak of war he was made senior medical officer to the *Monmouth* and presumably perished with that vessel. He was only thirty-five years of age.

Mr. Albert Joseph Tonkinson, the junior medical officer on H.M.S. *Monmouth*, graduated from the London Hospital by taking the M.B., B.S. Lond. only three years ago. He joined the Navy last year, but before doing so had gained a good deal of important experience as pathological assistant at the London Hospital and also as assistant medical officer to the St. John's Hill Infirmary at Wandsworth. He was likewise appointed to the *Monmouth* at the outbreak of war.

Mr. James Walsh graduated from the Irish schools in 1885 and entered the Naval Medical Service soon after qualification. He had been a fleet-surgeon for the last thirteen years and was appointed to the *Good Hope* last year. Just before this he had held the important post of medical officer to the Sheerness Dockyard and Sick Quarters.

Mr. Francis Charles Searle, who joined the *Good Hope* just previous to the outbreak of hostilities, was an old St. Bartholomew's Hospital man and had been resident medical officer to the Finsbury General Dispensary as well as prosector at the Royal College of Surgeons, being awarded a first-class certificate for his work there. Mr. Searle qualified M.R.C.S., L.R.C.P. in 1904 and subsequently took the M.B., B.S. Lond.

Mr. Fernand Louis J. M. de Verteuil came from the West Indies and studied medicine both at Edinburgh and London, where he was a student at King's College and St. Thomas's. Qualifying M.R.C.S., L.R.C.P. ten years ago, he ultimately proceeded to the M.D. degree of Edinburgh. Having for a time served in the Royal Navy, he passed into the Reserve, from which he was appointed to the *Good Hope* at the end of August. Previous to this Mr. de Verteuil had been practising at Vancouver, where he had found time to carry out important researches in connection with the bacteriology of syphilis, the use of salvarsan, and the pathology of yaws.

